# Whole-genome sequencing reveals genomic diversity and population structure of mango germplasm from the Jazan region of Saudi Arabia

**DOI:** 10.3389/fpls.2026.1808140

**Published:** 2026-05-01

**Authors:** Naser B. Almarri, Salwa M. Mostafa, Nada M Alsofuani, Sara Osman, Sara M. Alomran, Mohanad A. Ibrahim, Ibrahim Alrashidi, Omar A. Al-Haidar, Shakeel Ahmad

**Affiliations:** Ministry of Environment, Water and Agriculture (MEWA), Riyadh, Saudi Arabia

**Keywords:** *Mangifera indica*, whole-genome sequencing, population structure, genetic diversity, SNP fingerprinting, Jazan, Saudi Arabia

## Abstract

**Background:**

Mango (*Mangifera indica* L.) is an economically important tropical fruit crop, yet the genomic diversity of mango germplasm from the Jazan region of Saudi Arabia—the country's primary production hub—remains poorly characterized. This knowledge gap limits the effective use of regional genetic resources in breeding programs and conservation strategies under arid conditions.

**Methods:**

We conducted whole-genome resequencing of 64 mango accessions from the Jazan region. Reads were mapped to the *M. indica* reference genome, and variants were called. After stringent quality control and filtering, 5,079,569 high-quality SNPs were retained for genetic diversity and population structure analysis. A compact 150-SNP fingerprinting panel was subsequently designed for routine cultivar identification.

**Results:**

The genome-wide dataset revealed substantial genetic diversity (mean expected heterozygosity He = 0.3003, nucleotide diversity π = 5.87 × 10^-4^ per bp). Population structure analysis resolved three genetically differentiated ancestry groups (K = 3): a South Asian-associated cluster (n = 25), an admixed Saudi/Southeast Asian cluster enriched for local Jazan breeding selections (n = 19), and an Egyptian/Americas-associated cluster (n = 16). Pairwise FST values ranged from 0.048 to 0.089, indicating low-to-moderate differentiation with appreciable within-cluster diversity. Linkage disequilibrium decayed to r^2^ = 0.1 at approximately 142.5 kb. The 150-SNP fingerprinting panel distinguished all accessions with unique multilocus profiles (mean PIC = 0.3749, probability of identity = 5.25 × 10^-91^).

**Conclusion:**

Mango germplasm from the Jazan region harbors broad and structured genetic variation, establishing a regional genomic reference for accessions of diverse origin. The three-group structure reflects a complex introduction history with substantial admixture. The 150-SNP panel provides a practical tool for cultivar authentication, redundancy detection, and conservation prioritization, while the genome-wide SNP resource enables future trait-mapping studies and marker-assisted selection for mango improvement in tropical and subtropical dry regions.

## 1 Introduction

Mango is predominantly an outcrossing, heterozygous fruit tree that exhibits extensive genetic variation across its cultivars (Wang et al., 2020). Depending on their genetic origin, mango varieties produce either monoembryonic seeds (common in Indian cultivars) or polyembryonic seeds (typical of Southeast Asian cultivars) (Aron et al., 1998; Mukherjee and Litz, 2009; Wang et al., 2017). This standing genetic diversity is crucial for key agronomic traits; it underlies the spectrum of fruit qualities, disease resistances, and stress tolerances observed in mango, providing the raw material for crop improvement (Tanksley and McCouch, 1997; McCouch et al., 2013). Indeed, greater genetic variability offers breeders more opportunities to select for favorable traits and adapt the crop to new challenges (Tanksley and McCouch, 1997; McCouch et al., 2013).

Recent genomic advances have substantially enhanced our ability to characterize the genetic landscape of mango. High-quality, chromosome-scale genome assemblies for major cultivars (e.g., Alphonso and Tommy Atkins) now provide a robust foundation for fine-scale genetic analysis, trait mapping, and evolutionary studies (Wang et al., 2020; Varshney et al., 2021). These *de novo* reference genomes revealed that the mango genome is highly heterozygous (Wang et al., 2020), consistent with its outcrossing nature, and enabled the identification of genomic regions associated with domestication-related traits. For example, comparative genomics and selective sweep analyses have begun to pinpoint loci under selection for fruit quality attributes in perennial fruit crops (Duan et al., 2017). In one recent case, resequencing a broad mango germplasm panel uncovered multiple genetic clusters (corresponding largely to Indian vs. Southeast Asian germplasm) with little evidence of a single domestication bottleneck, and identified allelic variation in a key enzyme gene influencing fruit sugar content (Warschefsky and von Wettberg, 2019; Zhang et al., 2025).

In one recent case, resequencing a broad mango germplasm panel uncovered multiple genetic clusters (corresponding largely to Indian vs. Southeast Asian germplasm) with little evidence of a single domestication bottleneck, and identified allelic variation in a key enzyme gene influencing fruit sugar content (Warschefsky and von Wettberg, 2019; Zhang et al., 2025). Such findings underscore how genomic resources can illuminate the history of mango domestication and provide molecular targets for breeding. Molecular fingerprinting has become indispensable for cultivar identification, protection of breeding rights, and phytosanitary control in mango. Historically, growers have relied on morphological characters to distinguish cultivars; however, many fruit traits are plastic and environment-dependent, making purely phenotypic identification unreliable (Nadeem et al., 2018). Simple sequence repeat (SSR) markers and other DNA assays are widely used for cultivar authentication and germplasm diversity assessment, including in mango collections, owing to their co-dominance, multi-allelic profiles, and high polymorphism (Varshney et al., 2005). Studies leveraging highly polymorphic SSR panels consistently resolve substantial allelic diversity among cultivars and geographic structuring of gene pools, validating their utility for conservation and breeding (Ma et al., 2024). Collectively, these early marker-based surveys laid essential groundwork for today’s genome-wide SNP and resequencing approaches in horticultural crops, including mango (Wang et al., 2020).

Marker technologies have progressed from RAPD/AFLP to ISSR/SSR and now SNP arrays/sequencing, reshaping crop and fruit-tree genomics, including mango (Rasheed et al., 2017). NGS-derived SNPs provide dense, genome-wide markers for diversity analyses, association mapping, and MAS; reduced-representation methods (GBS, RAD/SLAF) and whole-genome resequencing enable rapid, cost-effective discovery even in complex genomes (Davey et al., 2011). Large resequencing projects uncover millions of SNPs and fine population structure, powering modern GWAS and genomic selection (Wang et al., 2018). In mango, chromosome-scale references now make genome-wide discovery routine for breeding, while ddRAD-seq efficiently yields thousands of SNPs to resolve population structure and phylogeography in non-model crops (Davey and Blaxter, 2010; Wang et al., 2020). In mango, ddRAD and related high-throughput genotyping methods have clarified the domestication and diversity of mango. RAD-seq reveals two major gene pools: the Indian monoembryonic and the Southeast Asian polyembryonic, without a severe dispersal bottleneck. Introductions drew on multiple sources, often boosting allelic diversity (Warschefsky and von Wettberg, 2019). Recent genomic analyses support a complex, multi-origin history with admixture and region-specific selection; one study resolved several clusters, proposed ≥2 domestication trajectories, and identified cell-wall invertase SNPs linked to extreme sweetness in a local landrace (Warschefsky and von Wettberg, 2019; Zhang et al., 2025).

Parallel advances in other long-lived fruit trees reinforce the notion that the domestication of perennial crops often produces mosaic genomes shaped by hybridization and repeated selection. In citrus, for instance, genome sequencing of diverse mandarins, pummelos, and oranges has revealed an intricate admixture network underlying the origins of cultivars (Wu et al., 2018). Wild and cultivated citrus species experienced reticulate evolution rather than a simple linear domestication, with introgressions (such as pummelo DNA in mandarin genomes) contributing to desirable fruit size and flavor characteristics (Wu et al., 2018). Likewise, in apple, large-scale resequencing of hundreds of accessions traced a two-stage domestication process involving initial selection in Central Asian *Malus sieversii*, followed by extensive introgression from wild European crabapples, culminating in the diverse array of modern apple cultivars (Duan et al., 2017). These genomic case studies illustrate how high-density markers and reference genomes can untangle complex domestication histories in clonal perennial crops and accelerate molecular breeding by identifying key trait loci. Similar approaches are now being applied to mango, which benefits from multiple reference genomes (e.g., Alphonso, Tommy Atkins, and Irwin) and an increasingly comprehensive SNP catalog. These resources have helped resolve phylogenetic relationships among cultivars, define the structure of global mango diversity, and associate genetic variants with important traits such as flowering time, fruit size, aroma, and sweetness (Duan et al., 2017; Wang et al., 2020; Zhang et al., 2025).

Despite rapid global progress in mango genomics, a comprehensive genome-wide characterization of mango germplasm from the Jazan region of Saudi Arabia remains limited. Landmark resources, chromosome-scale references and broad resequencing panels now resolve population structure and trait loci at high resolution, yet comparable datasets focused on Saudi cultivars are scarce (Wang et al., 2020). Previous global mango studies provide the main comparative framework for interpreting this panel. Warschefsky and von Wettberg (2019) analyzed a broad collection of mango accessions and showed that cultivated mango largely falls into two major gene pools corresponding to Indian and Southeast Asian germplasm, with no strong domestication bottleneck in introduced regions. More recent whole-genome resequencing studies extended this picture: Ma et al. (2024) confirmed the broad Indian-versus-Southeast Asian split but also resolved finer regional structure in Chinese and international germplasm, while Zhang et al. (2025) similarly recovered two major ancestry backgrounds together with substantial admixture and landrace-level substructure. Against this broader context, the Saudi/Jazan panel is informative not only as a regional case study but also as a test of how global mango ancestry components are represented, combined, and reshaped within germplasm cultivated in Saudi Arabia. Warschefsky and von Wettberg (2019) (Warschefsky and von Wettberg, 2019) reported two major cultivated gene pools and elevated diversity in introduced populations. (Ma et al. (2024) studied 224 accessions from 22 countries and found additional substructure beyond the two broad clades. Zhang et al. (2025) analyzed 90 accessions, recovered two major origin groups, and found widespread admixture plus local landrace structure.

The Jazan region in southwest Saudi Arabia is a nationally significant mango-producing zone, consistently cited as the country’s primary hub and leading contributor to national output (Ledesma et al., 2023). To date, the most recent Saudi study used fluorescent SSRs to genotype a limited set of local cultivars, offering sufficient but coarse resolution relative to genome-wide SNP approaches and underscoring the need for broader, higher-density surveys (Ahmed Safhi, 2024). Our objectives were to: (i) characterize the genetic diversity and population structure of mango germplasm from the Jazan region of Saudi Arabia in a global context; (ii) generate a validated SNP fingerprinting panel for precise cultivar identification and differentiation; and (iii) provide insights to inform mango breeding, conservation strategies, and the sustainable improvement of mango production under arid environmental conditions.

## 2 Materials and methods

### 2.1 Plant material and sampling

A total of 64 mango (*Mangifera indica* L., 2n = 40) accessions were collected from commercial farms in the Jazan region of southwestern Saudi Arabia and are maintained at the Seed Center and Plant Genetic Resources Bank (SC&PGRB) of the Ministry of Environment, Water and Agriculture (MEWA). A map showing the sampling location of all analyzed accessions in the Jazan region of southwestern Saudi Arabia is provided in **Supplementary Figure 1**. The panel includes several widely cultivated international cultivars such as ‘Nam Dok Mai K1’ (Thailand), ‘Carabao’ (Philippines), ‘Tommy Atkins’ and ‘Keitt’ (USA), ‘Fazli’ (Bangladesh), and ‘Julie’ (Jamaica), in addition to 21 new local breeding lines developed by Seed Centre as part of an ongoing mango improvement program. These local selections have not yet been formally named, released, or registered as cultivars and are therefore treated here as distinct germplasm entries under evaluation rather than as a single uniform cultivar group. Passport information (including local name, origin, and site descriptors) for all accessions is summarized in **Supplementary Table 1**. Throughout this study, the term “accession” refers to a catalogued germplasm entry in the collection, “cultivar” refers to a formally named variety, and “genotype” is used only in the molecular or genomic context. During the 2024 growing season, fully expanded, healthy leaves were sampled from mature trees. Leaf tissue was immediately placed in zipper-sealed plastic bags, transported on dry ice to the Plant Genomics and Biotechnology Laboratory (PGBL), and stored at −80 °C until DNA extraction.

### 2.2 DNA extraction and whole-genome sequencing

Genomic DNA was isolated from young leaf tissue using a modified CTAB method (Doyle and Doyle, 1987). Briefly, leaf samples were ground in liquid nitrogen to a fine powder and incubated in CTAB extraction buffer (2% CTAB, 100 mM Tris-HCl, 20 mM EDTA, 1.4 M NaCl, 0.2% β-mercaptoethanol) at 65 °C for 30 min. DNA was then purified using chloroform: isoamyl alcohol (24:1), precipitated with isopropanol, washed with 70% ethanol, and resuspended in TE buffer. DNA integrity was checked on agarose gels, while purity and concentration were assessed with a Nanodrop spectrophotometer, retaining samples with OD260/280 values between 1.8 and 2.2.

High-quality DNA (500 ng per sample) was used for library preparation at MajorBio (Shanghai, China) following the MGIEasy Universal protocol. DNA was fragmented with a Covaris system and size-selected to approximately 380–420 bp. Standard steps of end repair, A-tailing, adapter ligation, PCR amplification, purification, and quantification were applied. Library fragment sizes were examined on an Agilent 2100 Bioanalyzer. Single-stranded circular DNA was then generated to form DNA nanoball (DNB) arrays, and paired-end sequencing (150 bp) was carried out on the DNBSEQ-T7 platform (MGI Tech).

### 2.3 Read processing, alignment, and variant discovery

Raw reads (FASTQ files) from the 64 mango accessions were subjected to initial quality control using Fastp v0.23.2, which trimmed adapters, removed low-quality bases, and discarded reads with more than 40% of bases with a quality score (Q) below 20. The resulting clean reads were aligned to the *M. indica* reference genome ASM1674641v1 (GenBank accession GCA_016746415.1) using BWA-MEM v0.7.17 with default parameters. SAM files were converted to BAM format and sorted using SAMtools v1.13.

Variant calling was performed using GATK v4.1, and SNPs were retained with a minimum base quality of Q30. Subsequent filtering with VCFtools v0.1.16 and PLINK v1.9 was applied to obtain a high-confidence SNP dataset. Filters included mapping quality (MQ) > 37, quality-by-depth (QD) ≥ 24, minor allele frequency (MAF) > 0.05, and removal of loci with >20% missing data. The MQ threshold was set slightly below the commonly used GATK guideline of MQ > 40 (DePristo et al., 2011) to retain variants in repeat-adjacent regions of the mango genome while excluding poorly aligned sites. The QD threshold was set conservatively to retain only variants supported by strong per-read evidence, based on inspection of the raw variant-quality distribution under the sequencing depth obtained in this study.

### 2.4 Population genetic analyses

#### 2.4.1 Marker polymorphism and quality control

Population-level analyses were based on a stringently filtered SNP dataset. In PLINK v1.9, individuals with >20% missing genotypes (--mind 0.2) were excluded. SNPs with >5% missingness (--geno 0.05), those showing strong deviation from Hardy–Weinberg equilibrium (--hwe 1e-5), and loci with MAF < 0.05 (--maf 0.05) were removed. Linkage disequilibrium pruning was performed using the command --indep-pairwise 50 5 0.5. Samples with inbreeding coefficients (F) deviating by more than ±3 standard deviations from the mean were treated as heterozygosity outliers and excluded from downstream analyses.

#### 2.4.2 Population structure, principal component analysis, and phylogeny

The linkage-disequilibrium-pruned SNP set was used to infer population structure with ADMIXTURE v1.3.0. Values of K from 2 to 10 were tested, and the optimal K was determined based on the lowest cross-validation error, supported by the ΔK statistic. Individuals with ≥70% inferred ancestry from a single cluster were assigned to that cluster; the remaining accessions were classified as admixed. Ancestry proportions were visualized alongside geographical passport data using the ggplot2 package in R. Principal component analysis (PCA) was performed in PLINK v1.9 to summarize the major axes of genetic variation. Cluster robustness in the PCA space was evaluated using k-means clustering and silhouette scores in R.

To visualize overall genetic relatedness among accessions, we constructed a distance-based dendrogram from the LD-pruned SNP panel using pairwise identity-by-state (IBS) values. Genetic distance was defined as 1 − IBS, and hierarchical clustering was performed using the unweighted pair-group method with arithmetic mean (UPGMA). Because the collection includes admixed genotypes and no suitable outgroup was available, this analysis was used as a complementary visualization of relatedness and cluster structure, rather than as a literal reconstruction of branching history. Concordance among IBS-based clustering, ADMIXTURE, and PCA was examined to obtain a coherent picture of the population structure. In addition to ancestry inference, we calculated cluster-specific observed heterozygosity (Ho), expected heterozygosity (He), pairwise weighted FST, and nucleotide diversity (π) for the three ADMIXTURE-defined groups using the curated SNP dataset. We also evaluated linkage disequilibrium (LD) decay from the full, non-LD-pruned SNP set by examining the decline of pairwise r^2^ with physical distance across the genome.

### 2.5 Construction of SNP fingerprinting panel and 2D barcodes

A custom SNP fingerprinting panel was designed to support accurate cultivar identification and germplasm management. From the high-quality SNP dataset, we selected bi-allelic markers with low linkage disequilibrium (pairwise r^2^ < 0.2), minor allele frequency (MAF) > 0.25, polymorphic information content (PIC) > 0.25, and heterozygote informativeness criteria (P(AB) > 0.30). This filtering yielded 150 highly informative SNPs. Selection was intentionally constrained to retain markers with broad genome representation and to minimize redundancy among tightly linked loci. Markers were further selected to achieve an approximately even distribution across the 20 mango chromosomes, thereby maximizing overall discriminatory power. Genotypic profiles for the 64 accessions, based on 150 SNPs, were summarized as accession-by-marker heatmaps for visual comparison. For each accession, the unique 150-SNP genotype was encoded as a Quick Response (QR) code using the online QR Code Generator tool (http://goqr.me/). This system enables rapid cultivar verification in field and trade environments by scanning QR codes, providing a practical framework for germplasm conservation, management, and protection of cultivar identity.

## 3 Results

### 3.1 Sequencing, variant discovery, and dataset quality

**Figure 1:**
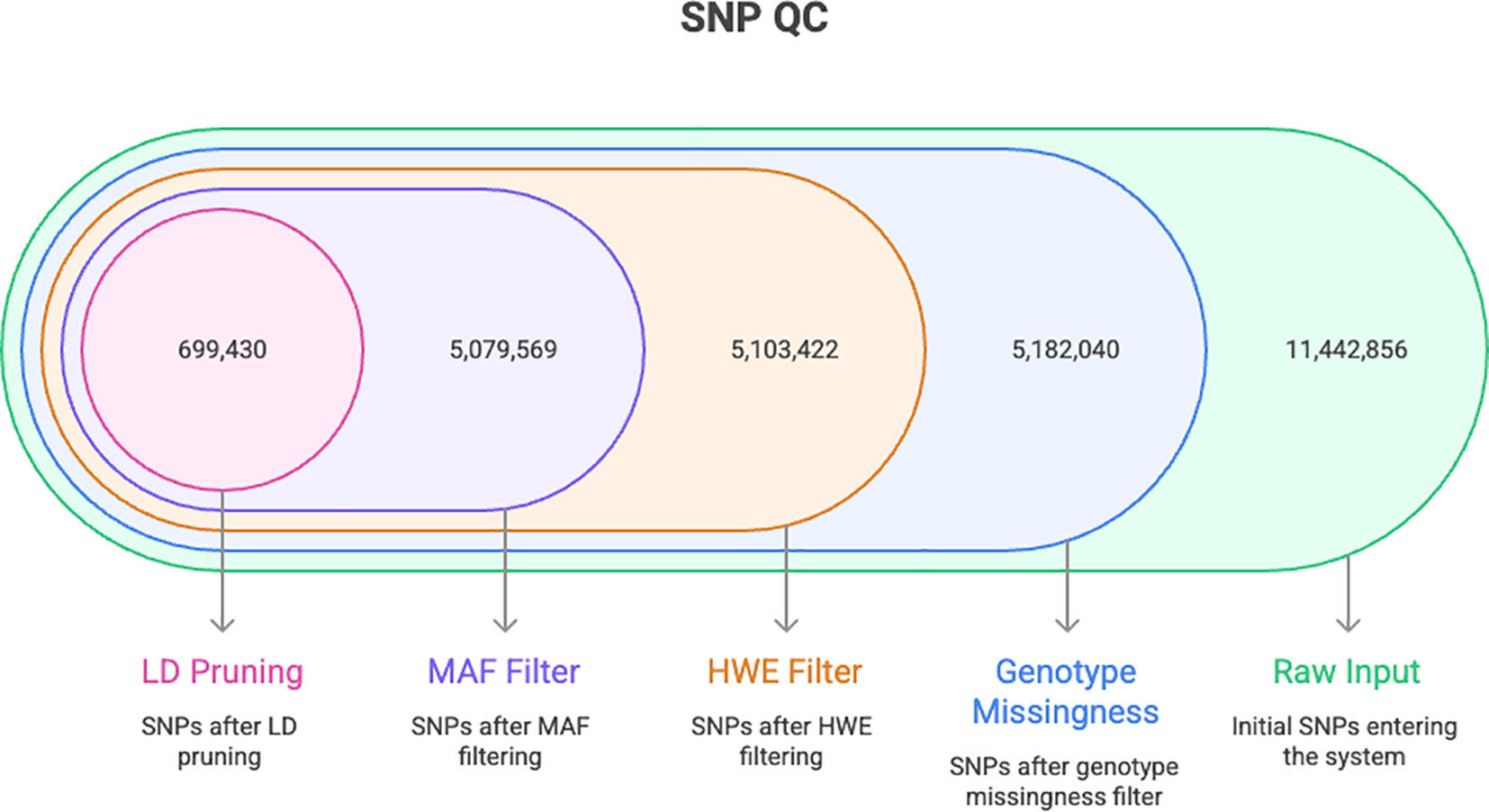
Summary of sample and variant attrition across the quality-control pipeline, from raw SNP discovery through site-level filtering, LD pruning, and final sample curation. The filtering procedure retained 60 high-quality accessions and 699,430 LD-pruned SNPs, providing a robust dataset for downstream population-genomic analyses.

Whole-genome sequencing of 64 *Mangifera indica* accessions yielded 12.6-20.7 million reads per sample, corresponding to 3.8–6.2 Gb per accession. After read cleaning, quality remained uniformly high, with Q30 values of 95.1–97.4% and GC content of 32.7–36.6% (**Figure 1**; **Supplementary Table 2**). The reads were aligned to the *M. indica* reference genome (GCA_016746415.1), and most libraries showed mapping rates above 93% with proper-pairing rates exceeding 88%, indicating consistent library preparation and reliable mappability. Initial variant discovery across all accessions identified 11,442,856 SNPs. We first assessed per-sample genotype completeness using a genotype missingness threshold of 0.2, which led to the removal of two accessions with excessive missingness: M38 (Mulgoba, USA; 50.31%) and M85 (Zill, USA; 28.43%). The working cohort, therefore, comprised 62 accessions at this stage, and all 11,442,856 discovered SNPs were retained for site-level quality control (**Supplementary Figure 2**).

**Figure 2:**
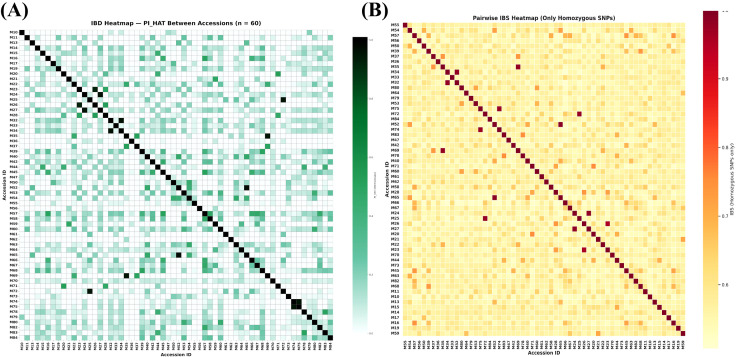
Identity-by-descent **(A)** and identity-by-state **(B)** analyses among 60 mango accessions. No accession pair met thresholds consistent with duplication or very close relatedness, supporting the genetic distinctness of the curated panel.

Variant-level filtering proceeded in three steps. Filtering on site missingness at a 5% threshold removed 6,261,816 SNPs, leaving 5,181,040 sites for further evaluation. We then excluded 38,618 SNPs that deviated from the Hardy–Weinberg equilibrium at P < 1 × 10^(-5), retaining 5,142,422 sites. Finally, variants with minor allele frequency below 0.05 (62,853 SNPs) were removed, producing a set of 5,079,569 high-quality common SNPs across the 62 accessions. Thus, ~44% of the initially discovered variants passed these completeness, equilibrium, and frequency criteria and were used as the basis for downstream analyses. The chosen filtering thresholds were intended to balance stringency and marker retention and did not introduce anomalous patterns in downstream PCA, ADMIXTURE, or relatedness analyses. To obtain an approximately independent marker panel suitable for population-genetic inference, we applied linkage-disequilibrium pruning with a 50-SNP window, a 5-SNP step, and an r2 threshold of 0.5. This step eliminated 4,380,139 linked variants and yielded 699,430 LD-pruned SNPs. We first assessed per-sample genotype completeness using a genotype-missingness threshold of 0.2, which led to the removal of two accessions with excessive missing data: M38 (Mulgoba, USA; 50.31%) and M85 (Zill, USA; 28.43%). We next examined sample-level heterozygosity using the inbreeding coefficient (F). Most accessions fell within the expected range for the panel; however, two individuals, M30 (a new selected variety from Saudi Arabia) and M51 (Anb-elyaman, Yemen), were more than 3 standard deviations from the mean and were excluded as heterozygosity outliers (**Supplementary Figure 3**). The excluded accessions therefore represent more than one passport group and geographic origin, suggesting that these QC-based removals are unlikely to reflect systematic bias against a single germplasm class. The final analysis set, therefore, consisted of 60 high-quality accessions typed at 699,430 LD-pruned SNPs. We further evaluated potential redundancy or cryptic relatedness using identity-by-state and identity-by-descent metrics. No pair of accessions met commonly used thresholds for near-identity (IBS ≥ 98%) or duplicate/first-degree relatedness (PI_HAT > 0.98), confirming the absence of duplicated or closely related entries in the curated panel (**Figure 2**).

**Figure 3:**
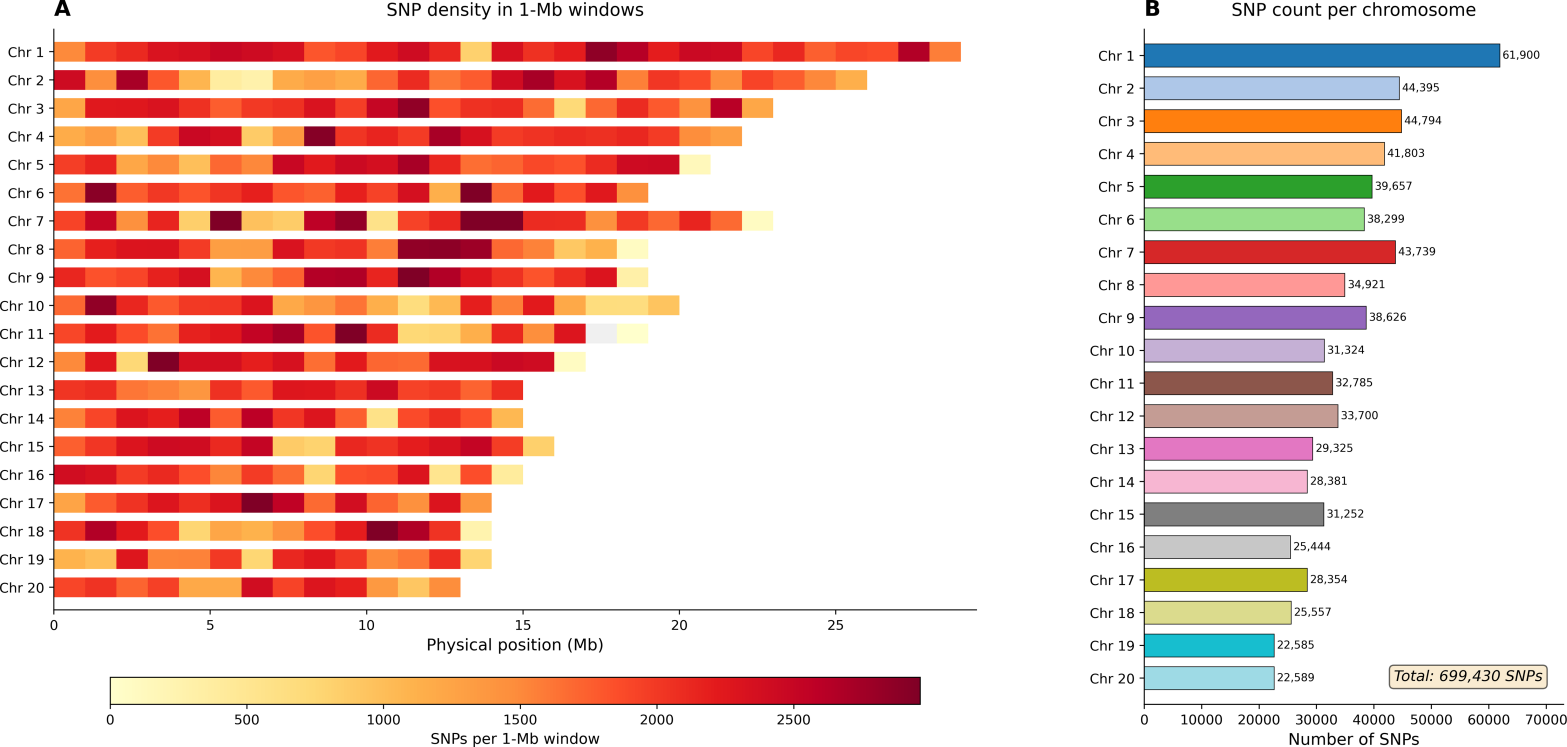
Genome-wide distribution of 699,430 high-quality SNPs across the 20 *Mangifera indica* chromosomes following quality control and LD pruning. **(A)** Heatmap of SNP density in non-overlapping 1-Mb windows along each chromosome, with color intensity (YlOrRd gradient; range 0 to ~2,800 SNPs per window) indicating SNP density from low (light) to high (dark). Chromosomes are ordered from 1 to 20 (top to bottom) and scaled according to physical length (Mb). **(B)** A bar plot of total SNP counts per chromosome shows an uneven SNP distribution across the genome.

The 699,430 LD-pruned SNPs were broadly and evenly distributed across the 20 chromosomes of the *M. indica* reference, with chromosome-level counts tracking physical length. Chromosome 1 carried the largest share of markers (61,900 SNPs), whereas chromosome 19 contained the fewest (22,585 SNPs), a pattern consistent with differences in chromosome size and local genomic organization. Along each chromosome, SNP density was summarized in non-overlapping 1-Mb windows, revealing a generally uniform landscape punctuated by localized troughs and peaks that likely reflect repetitive/low-complexity regions, variable gene density, or assembly gaps. The resulting density heatmap is plotted against physical distance and color-scaled by the number of SNPs per 1-Mb bin, providing a visual check that coverage remained even at chromosome ends and in interstitial regions (**Figure 3**; **Supplementary Table 3**).

Average sequencing depth across samples was ~12.5×, which is sufficient for confident genotype calling and downstream population analyses (**Supplementary Table 4**). In combination with the near-uniform chromosomal spread of variants, this depth minimizes ascertainment bias. It supports robust inference of diversity and structure (e.g., PCA, admixture, kinship), as well as LD-aware analyses that benefit from evenly spaced markers. Together, these features indicate that the final SNP panel provides comprehensive genome coverage at a resolution appropriate for population-genomic and association studies in *M. indica*. Linkage disequilibrium (LD) decay was additionally evaluated using the full, non-LD-pruned SNP dataset. Genome-wide LD declined with increasing physical distance, with mean r^2^ showing a half-decay distance of 39.4 kb and decreasing to 0.1 at approximately 142.5 kb (**Supplementary Figure 4**). These results indicate moderate LD persistence across the panel and support the use of LD pruning for downstream population-genetic analyses.

### 3.2 Population structure

**Figure 4:**
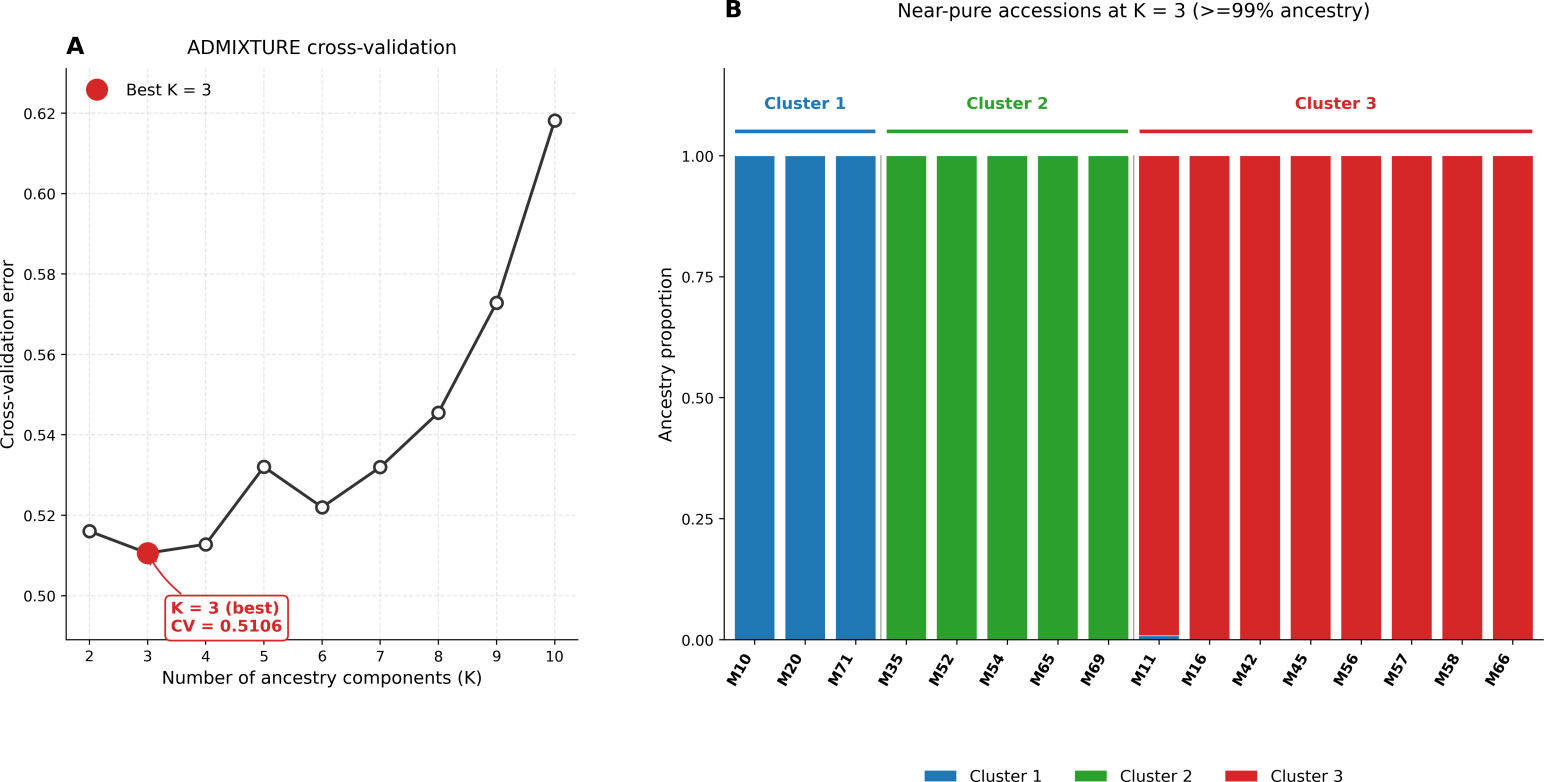
ADMIXTURE model evaluation for the 60 curated mango accessions. **(A)** Cross-validation error plot identifying K = 3 as the best-supported number of ancestry groups. **(B)** Representative near-pure accessions were assigned to each of the three inferred ancestry clusters.

To characterize the population structure of the curated panel, we analyzed 699,430 LD-pruned SNPs from 60 Mangifera indica accessions using ADMIXTURE. Model complexity was evaluated using cross-validation for K values ranging from 2 to 10. The cross-validation error showed a clear minimum at K = 3 (CV = 0.51057). The CV errors at K = 2 and K = 4 were only slightly higher, whereas the error increased further from K ≥ 5, supporting K = 3 as the most appropriate model for this dataset (**Figure 4A**). For transparency, the CV errors for K = 2–5 are shown in **Supplementary Figure 5**. Using the K = 3 solution, individuals were assigned to clusters based on their maximum ancestry coefficient (Q). We adopted a strict criterion, Q ≥ 0.99, to flag genetically pure representatives of a cluster and treated the remainder as admixed to varying degrees. By this definition, 16 accessions were identified as near-pure, including M54, M56, M57, M52, M65, and M69, each showing negligible contributions from alternative components; the remaining accessions exhibited graded admixture consistent with historical gene flow and cultivar exchange (**Figure 4B**). The K = 3 model resolved three biologically interpretable groups that broadly mirror known geographic and cultivar histories. Cluster 1 comprised 19 accessions (~43.3%) and exhibited the highest admixture, with individuals carrying appreciable proportions of two or all three components. This cluster included many of the Jazan local breeding selections and several introductions from Thailand, Indonesia, and South Africa. Because these Jazan local breeding selections represent heterogeneous, not-yet-registered material rather than a single uniform cultivar class, the elevated admixture observed in this cluster is consistent with mixed ancestry and/or multiple introduction histories within this material. Representative members included M16, M36, M59, and M66, and several accessions in this group retained substantial secondary ancestry fractions, indicative of either recent intercrossing or shared ancestry among donor lineages.

Cluster 2 contained 25 accessions (~35%) and was characterized by strong assignment to a single component for most individuals, reflecting a comparatively homogeneous genetic background. This group was dominated by accessions of South Asian origin, cultivars from India, Pakistan, and Bangladesh, with a smaller subset of Saudi material that shares the same ancestral signature. Examples include M10, M11, M20, and M45. Although our stringent Q ≥ 0.99 threshold identified only a subset of pure individuals across the entire data set, the majority of Cluster 2 samples nonetheless showed high (>0.90) membership in the same component, in contrast to the heterogeneous profiles observed in Cluster 1. Cluster 3 comprised 16 accessions (~21.7%) and, like Cluster 2, was dominated by a single ancestry component. This set included several Egyptian cultivars, as well as introductions from the Americas, such as Palmer, Van Dyke, Tommy Atkins, and Keitt, which formed a cohesive group with limited secondary ancestry. Illustrative members were M13, M26, M40, M52, and M73. The distinctiveness of Cluster 3 suggests a breeding or domestication trajectory that is partially independent of the South Asian group and largely separate from the admixed Saudi and Southeast Asian material. Accordingly, the three inferred groups do not map one-to-one onto Indian, Southeast Asian, and a residual ‘other’ pool; instead, they represent a South Asian-associated group, a Saudi/Southeast Asian admixed group, and an Egyptian/Americas-associated group. Across the full LD-pruned genome-wide dataset, 699,430 SNPs were retained for population analysis. These loci showed a mean minor allele frequency (MAF) of 0.2151 and a mean polymorphic information content (PIC) of 0.2463. The mean expected heterozygosity across loci was 0.3003, indicating substantial genome-wide diversity within the panel. Under the K = 3 ADMIXTURE solution, the 60 curated accessions were assigned to three ancestry groups comprising 19, 25, and 16 accessions, respectively. Additional population-genomic statistics further supported the three-group structure. Observed heterozygosity (Ho) was 0.305 in Cluster 1 (n = 19), 0.312 in Cluster 2 (n = 25), and 0.286 in Cluster 3 (n = 16), while expected heterozygosity (He) was 0.300 in all three clusters. Pairwise weighted FST values indicated low-to-moderate differentiation, with the highest differentiation observed between Clusters 1 and 3 (FST = 0.089), followed by Clusters 2 and 3 (FST = 0.065) and Clusters 1 and 2 (FST = 0.048). Genome-wide nucleotide diversity (π) across all 60 accessions was 5.87 × 10^−4^ per bp, with cluster-specific values of 5.58 × 10^−4^, 5.78 × 10^−4^, and 5.41 × 10^−4^ per bp for Clusters 1, 2, and 3, respectively (**Supplementary Figure 6**). These results indicate appreciable within-cluster diversity together with measurable genetic differentiation among the inferred ancestry groups (**Supplementary Table 5**). Although these summary metrics provide a clearer quantitative description of differentiation and diversity in the panel, broader sampling and denser within-region comparisons will be valuable in future work to refine these estimates across a wider representation of mango germplasm.

Across the panel, several accessions (e.g., M28, M44, and M71) exhibited intermediate membership coefficients, consistent with either recent introgression events or long-standing admixture among cultivar groups. Interpreted in conjunction with the LD-pruned marker set (which minimizes correlation among loci) and the stringent assignment rule, these results suggest that the collection encompasses three major ancestry backgrounds, characterized by uneven but interpretable admixture in a subset of samples. The cross-validation profile (**Figure 3A**) and the bar plot of ancestry coefficients (**Figure 4A**) collectively support K = 3 as the parsimonious model for this material, while leaving open the possibility that finer substructure could emerge within clusters if additional sampling from particular regions or breeding programs were included.

PCA performed on the LD-pruned panel of 699,430 SNPs in 60 *Mangifera indica* accessions provided an independent line of evidence for the population structure inferred with ADMIXTURE. The first two principal components captured a substantial fraction of the total standing variation (PC1 = 19.13%, PC2 = 13.71%; cumulative = 32.84%), revealing three prominent groupings in the ordination space. Accessions assigned by ADMIXTURE to a single ancestry component (Q ≥ 0.99) tended to occupy the extremes of the scatter along PC1 and PC2. In contrast, individuals with appreciable admixture plotted between these extremes, forming graded transitions rather than distinct outliers. This geometry is consistent with allele-frequency differences among the three inferred ancestry backgrounds and with historical gene flow among cultivar groups.

**Figure 5:**
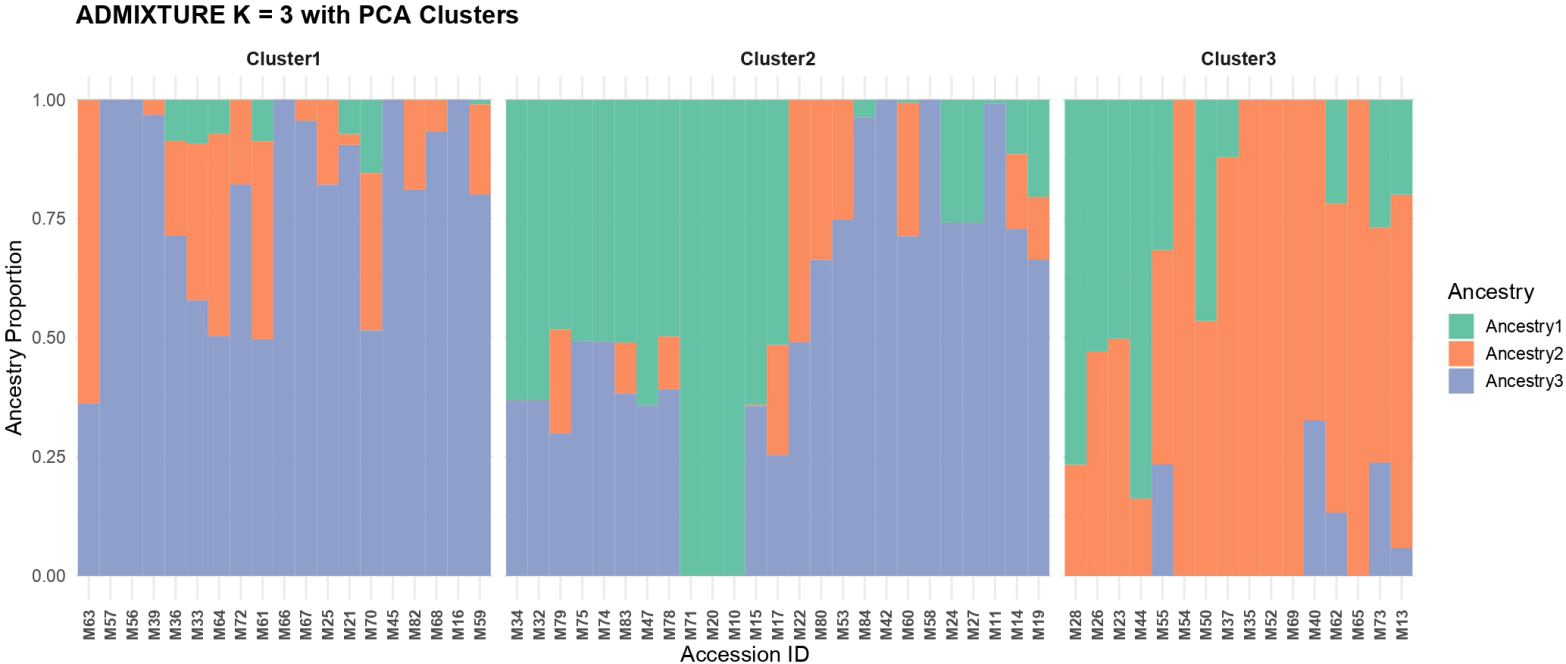
ADMIXTURE bar plot of 60 mango accessions at K = 3. Each bar represents one accession, and the three colors indicate proportional ancestry from the inferred clusters. The plot highlights both clearly assigned accessions and admixed accessions, revealing substantial ancestry mixing within part of the collection.

The spatial arrangement of points in the biplot broadly mirrored the ADMIXTURE partitions: one lobe comprised largely homogeneous accessions of South Asian provenance, a second lobe contained cultivars of Egyptian/Americas origin, and a third, more diffuse grouping encompassed Jazan local breeding selections together with several Southeast Asian introductions. The modest overlap observed at the boundaries among clusters reflects shared ancestry in admixed accessions and aligns with the intermediate Q-values in the ADMIXTURE bar plot. Importantly, no single accession dominated the dispersion pattern, and the absence of strong outliers supports the earlier data curation steps (sample-level missingness and heterozygosity filtering). Taken together, the concordance between the PCA ordination and the K = 3 ADMIXTURE model strengthens the inference that our panel comprises three genetically differentiated ancestry groups with varying degrees of admixture, providing a coherent framework for downstream analyses of diversity, relatedness, and trait association in mango (**Figure 5**).

**Figure 6:**
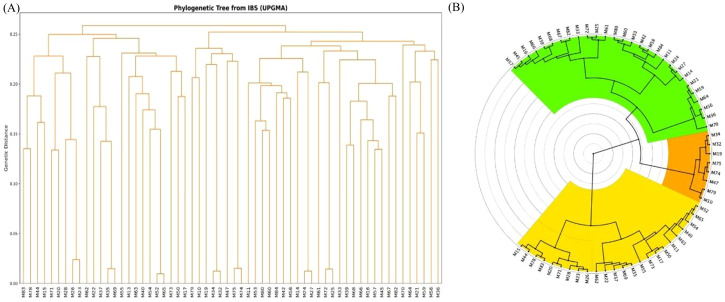
IBS-based UPGMA clustering of the 60 curated mango accessions. **(A)** Rectangular dendrogram constructed from pairwise identity-by-state (IBS) distances, where genetic distance = 1 − IBS. **(B)** Circular representation of the same clustering. The tree resolves three broad relatedness groups and supports the absence of near-duplicate accessions, while also showing that some accessions occupy intermediate positions consistent with admixture.

Overall genetic relatedness among the 60 curated Mangifera indica accessions was visualized using a distance-based dendrogram constructed from identity-by-state (IBS)–derived distances (1 − IBS) and clustered using the unweighted pair-group method with arithmetic mean (UPGMA) (**Figure 6**). The resulting dendrogram shows clear separation among lineages and an absence of extremely short terminal branches, indicating that no accession is a near-duplicate or clonally derived copy of another. This conclusion is consistent with our pairwise relatedness screen, in which no accession pair exceeded commonly accepted redundancy thresholds (IBS ≥ 98% or PI_HAT > 0.98), reinforcing that the panel represents distinct genotypes rather than replicated or very closely related samples. The overall topology mirrors the population structure recovered by ADMIXTURE and PCA, with three broad clades corresponding to the major ancestry backgrounds and admixed individuals positioned on intermediate or unusually long paths. Several accessions, such as M11, M52, M54, and M66, occur at the tips of long, solitary branches and display dominant membership in a single ADMIXTURE component. Their placement is consistent with relatively unadmixed lineages that have accumulated allele-frequency differences from the rest of the panel. In contrast, accessions previously flagged as admixed (e.g., M37, M45, M66, and M68) occupy long, distinctive branches that connect near internal nodes, a pattern expected when genomes carry sizeable fractions of ancestry from multiple sources. These branch-length patterns, along with their mosaic ADMIXTURE profiles, indicate substantial genomic divergence accompanied by historical introgression. Because many accessions show admixture, the dendrogram should be interpreted primarily as a summary of pairwise relatedness and cluster structure, rather than as a strict bifurcating evolutionary history.

**Figure 7:**
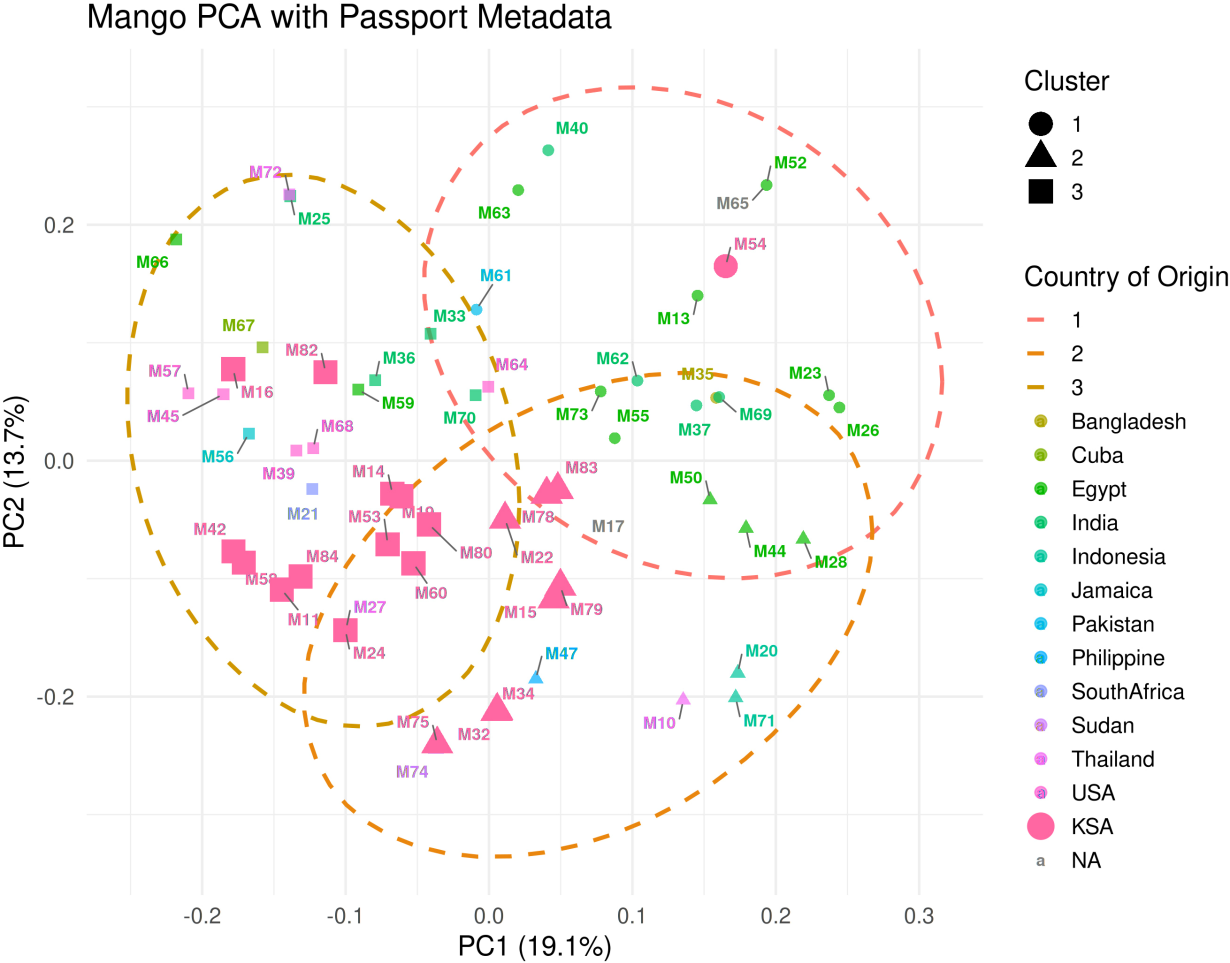
Principal component analysis (PCA) of 60 *Mangifera indica* accessions. The first two principal components explain 19.13% and 13.71% of the total genetic variance, respectively, and resolve three main groups consistent with the ADMIXTURE and IBS-based clustering results, with admixed accessions occupying intermediate positions.

Subclade structure is also apparent within the major groups. For example, accessions M24, M25, M26, M27, M35, and M69 cluster within the same broader subgroup yet retain discernible terminal branch lengths, demonstrating that, although clearly more similar to one another than to the rest of the panel, they are not redundant entries. Additionally, smaller clusters exhibit comparable spacing among tips, suggesting a shared breeding history or geographic provenance within clades, while maintaining sufficient divergence to justify their inclusion as unique accessions. Taken together, the tree corroborates the existence of three principal genetic lineages in our collection and clarifies how admixed accessions bridge these groups. Across the final 150 loci, PIC averaged 0.3749 (range 0.3747–0.3750), observed heterozygosity averaged 0.2747 (range 0.2105–0.2982), and per-locus missing data averaged 3.63% (range 0.0–5.0%). Under approximate locus independence following LD pruning, the cumulative probability of identity was 5.25 × 10^−91^ and the probability of identity among siblings was 1.12 × 10^−34^, indicating extremely high fingerprinting power. Together with the unique multilocus profiles observed for all 60 accessions and the broad chromosomal distribution of the source SNPs, these results support the use of the panel for routine accession verification and germplasm management. The concordance among UPGMA topology, ADMIXTURE assignments, and PCA scatter further supports the robustness of our curation and the suitability of this panel for downstream analyses of diversity, relatedness, and trait association (**Figure 7**).

### 3.3 A 150-SNP fingerprinting panel for unambiguous accession identification

**Figure 8:**
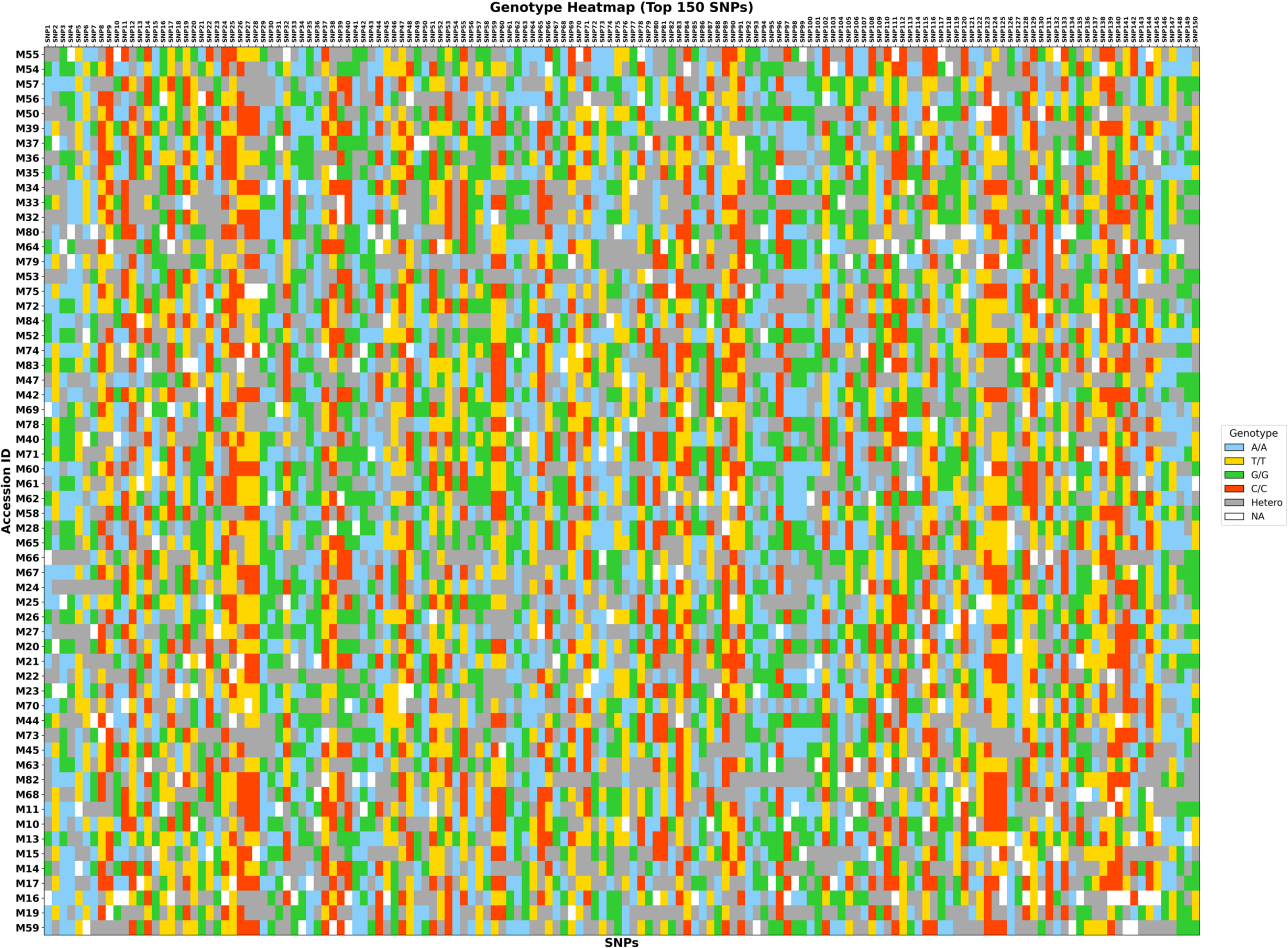
Genomic fingerprints of 60 *Mangifera indica* accessions based on the 150-SNP panel. Rows correspond to accessions and columns to loci; homozygotes are color-coded, heterozygotes are grey, and missing calls are white. Each accession shows a unique multilocus profile, demonstrating the discriminatory power of the fingerprinting panel within the curated dataset.

To enable unambiguous identity verification and routine curation of the collection, we distilled the variant set to a compact fingerprinting panel of 150 highly informative SNPs. Candidates were selected from the LD-pruned genome-wide dataset and prioritized based on polymorphism (high minor-allele frequency and heterozygosity), low missingness, and physical spacing to minimize the impact of tightly linked markers. Preference was given to sites that were consistently genotyped across accessions and that retained discrimination power within and among the ADMIXTURE-defined clusters, thereby maximizing resolution without sacrificing robustness. The resulting multilocus profiles were visualized as a genotype heatmap, with rows corresponding to accessions and columns to SNP loci, and homozygotes coded by nucleotide-specific colors (A/A, C/C, G/G, T/T), heterozygotes in grey, and missing calls in white (**Figure 8**; **Supplementary Table 8**). Across the 60 accessions, every multilocus profile was unique; no two entries shared an identical 150-SNP signature, confirming that the panel has sufficient information content for accession-level identification. However, this validation was performed within the same curated dataset from which the panel was derived and therefore demonstrates internal discriminatory power rather than independent external validation. The heatmap also reveals orderly blocks of heterozygosity and allele sharing that mirror the population structure inferred from ADMIXTURE and PCA. At the same time, the very low frequency of white cells attests to the high call rate of the selected loci. In addition, the fingerprints of these accessions exhibited rich variation and were encoded as a 2D barcode accessible via a cell phone (**Supplementary Figure 7**).

**Figure 9:**
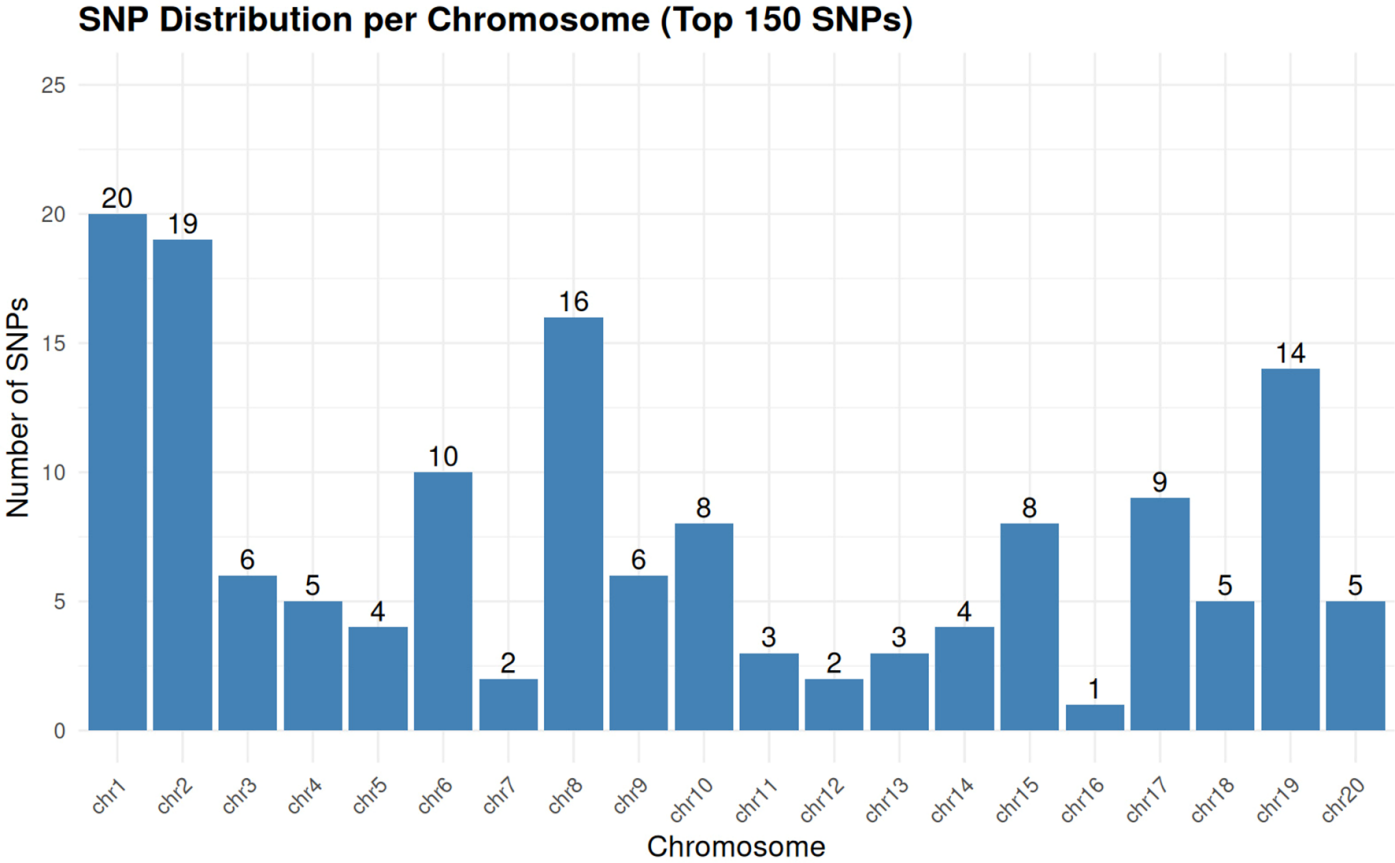
Chromosomal distribution of the 150 SNPs included in the fingerprinting panel. Markers are distributed across all 20 chromosomes, indicating broad genome representation and supporting the robustness of the panel for accession identification.

The chromosomal distribution of these 150 SNPs spans all 20 *M. indica* chromosomes, providing genome-wide coverage suitable for routine DNA fingerprinting and parentage and mislabel detection (**Figure 9**). Marker counts per chromosome generally tracked physical length and background polymorphism: chromosomes 1 and 2 contributed the largest numbers (20 and 19 SNPs, respectively), whereas chromosomes 7 and 16 contributed fewer sites (2 and 1 SNPs). This non-uniformity reflects intentional down-weighting of redundant, high-LD regions and scarcity of high-quality, high-MAF sites in some intervals, rather than genotyping failure, and it preserves broad coverage with an average of ~7–8 markers per chromosome. Together, the even genome representation, high per-locus performance, and complete distinctness of multilocus genotypes demonstrate that this 150-SNP panel provides a practical, cost-effective barcode for *M. indica* germplasm. The final 150 loci are biallelic SNPs suitable for conversion to routine allele-specific genotyping platforms such as KASP or TaqMan; exact per-sample cost depends on provider, chemistry, and batch size, but low-density KASP services may start at about £1.75 per sample, whereas TaqMan implementation is typically more reaction-dependent and should be costed per assay design and throughput.

## 4 Discussion

Our whole-genome resequencing produced a dense, high-quality variant catalogue that remained robust after stringent filtering, consistent with best-practice pipelines for short-read variant discovery and cohort genotyping. High Q30 values and mapping rates (**Figure 1**) are in line with community standards that link base-quality calibration, local realignment, and cohort joint-calling to accurate SNP sets in population studies. They mirror widely adopted frameworks such as GATK and its successors (e.g., DeepVariant), which have shown strong cross-platform performance and scalability to large cohorts (DePristo et al., 2011). Our SNP- and sample-level QC thresholds (per-marker missingness, HWE, and MAF, plus sample missingness and heterozygosity) reflect established recommendations for avoiding artefacts and spurious inference in population analyses (**Figure 1**), and align with widely cited QC protocols by Anderson et al (Anderson et al., 2010). The final LD-pruned working set (699,430 SNPs) provides the unlinked markers required by model-based clustering and PCA, a design choice broadly recommended in method papers and large-scale structure analyses (Alexander et al., 2009).

The broad genome coverage of the retained SNP set is consistent with chromosome-scale mango genomic resources and supports the reliability of downstream diversity and structure inferences. In those datasets, SNP density generally covaries with gene density, repeats, and GC content. Recent mango assemblies and resequencing studies, spanning a reference genome, population resequencing, and an updated gapless assembly, likewise report broad, genome-wide SNP distributions that track chromosomal attributes and recent genome history (Wang et al., 2020). These genomic features in mango mirror observations across other fruit-tree genomes, in which chromosome architecture, transposable element landscapes, and historical duplication events shape polymorphism landscapes. Examples include citrus, peach, pear, banana, grape, and domesticated apple (used here as tree-crop comparators) (Wu et al., 2018). Together, these benchmarks support the conclusion that our depth (~12.5×) and breadth of coverage were adequate for confident variant discovery and downstream population inference in a perennial tree species (DePristo et al., 2011). Model-based clustering supported a three-group structure with one notably admixed component, consistent with repeated introduction, exchange, and recombination among mango accessions cultivated in the region. Additional population-genomic summary statistics further support the structure inferred from the genome-wide SNP dataset. Cluster-specific observed heterozygosity (Ho) ranged from 0.286 to 0.312, pairwise weighted FST ranged from 0.048 to 0.089, and nucleotide diversity (π) ranged from 5.41 × 10^−4^ to 5.78 × 10^−4^ per bp among the three clusters, while the full panel showed a genome-wide π of 5.87 × 10^−4^ per bp. In addition, LD decay based on the full SNP dataset showed a half-decay distance of 39.4 kb, with mean r^2^ declining to 0.1 at 142.5 kb. Together, these values indicate appreciable within-group diversity and low-to-moderate differentiation among the inferred ancestry groups. Notably, much of the admixed group includes accessions representing local breeding selections developed by Seed Centre, which in this study are treated as heterogeneous, not-yet-registered materials rather than as a single named cultivar. Accordingly, these accessions should be managed as distinct accession-level breeding and conservation resources until further phenotypic evaluation and formal classification are completed. This pattern is expected in clonally propagated, outcrossing tree crops that have experienced region-specific selection, recurrent hybridization, and movement through trade over centuries. Comparable admixture mosaics and regional clades have been reported in mango resequencing panels and in high-quality mango genomes that reconstruct domestication and diffusion histories (Warschefsky and von Wettberg, 2019). Viewed against those broader studies, our K = 3 result only partially aligns with the simple Indian-versus-Southeast Asian framework. In our panel, Cluster 2 corresponds most closely to a South Asian-associated ancestry group. In contrast, Cluster 1 contains a more admixed Saudi/Southeast Asian assemblage enriched for Jazan local breeding selections and introductions from Thailand and Indonesia. Cluster 3 is not merely an undifferentiated ‘other’ group, but is enriched for Egyptian and Americas-origin cultivars, suggesting that the Saudi collection captures an additional layer of introduction-driven and regionally maintained structure. Thus, the Jazan/Saudi dataset both recovers the broad gene-pool signal described in global mango studies and reveals how those ancestry components are reorganized within germplasm circulating in a major Arabian production system. A key limitation of this study is its geographic scope. All analyzed accessions were collected from the Jazan region of southwestern Saudi Arabia, and therefore the dataset represents mango germplasm from this region rather than the full diversity of mango cultivated across Saudi Arabia. Accordingly, the population structure and diversity patterns reported here should be interpreted as a regional genomic baseline for Jazan mango germplasm, not as a complete national representation. Broader sampling across other mango-growing regions of Saudi Arabia will be necessary in future studies to assess country-wide diversity. The global reference studies consistently recover broad Indian and Southeast Asian ancestry divisions, while larger recent mango genomic panels also reveal finer regional structure and admixture. Our results are broadly consistent with this pattern: the Jazan panel retains the major ancestry signal described in global mango studies but also shows additional regional structuring associated with germplasm maintained in southwestern Saudi Arabia. The observed low-to-moderate pairwise differentiation among clusters and the substantial within-cluster heterozygosity further support the view that this collection reflects a history of multiple introductions, admixture, and local maintenance rather than isolation into sharply separated lineages. At the same time, direct benchmarking against global mango datasets remains limited because only a small number of large-scale whole-genome studies currently report fully comparable population-genomic summary statistics. This highlights the need for broader, harmonized mango genomic resources to enable more comprehensive cross-study comparisons in the future.

Methodologically, our ADMIXTURE implementation with cross-validation follows the original maximum-likelihood framework, which is optimized for large SNP datasets. The use of LD-pruned SNPs is consistent with this framework’s assumptions (Alexander et al., 2009). Independent ordination by PCA corroborated the same broad structure, strengthening confidence that the inferred groups reflect underlying biological relationships rather than analytical artefacts. The agreement between ADMIXTURE and PCA strengthens the inference of three genetic ancestry groups while highlighting admixed individuals, a convergence routinely observed in human and plant datasets when PCA is used both to visualize structure and to diagnose stratification (Price et al., 2006). We further verified the absence of duplicates or clonal replicates through IBS/IBD screens (**Figure 2**), applying widely used thresholds and software, which is standard practice before downstream analyses (Purcell et al., 2007). The IBS-based UPGMA dendrogram (**Figure 6**) complements these results by providing a simple distance-based visualization of overall relatedness among accessions. Its broad grouping pattern is consistent with the ADMIXTURE and PCA results and with the historical picture emerging from mango genomics, which suggests multiple introductions, regional diversification, and occasional interlineage introgression (Wang et al., 2020). However, because several accessions are admixed, the dendrogram is interpreted here as a complementary summary of genetic similarity rather than as a literal branching history. The genomic structure observed in this panel may also have implications for adaptation to arid production environments. Mango cultivation in the Jazan region occurs under hot and relatively dry conditions, and the distinct clustering of locally maintained material suggests that selection under these conditions, together with breeding history, may have contributed to shaping the diversity patterns detected here. In particular, the cluster enriched for local Jazan breeding selections showed slightly lower observed heterozygosity than the other two groups, which may be consistent with selection during local adaptation or with repeated use of related breeding material. This interpretation should remain cautious, however, because the present study was not designed to test adaptive loci directly. Future work integrating genomic, phenotypic, and environmental data will be needed to determine whether the structure observed here reflects adaptation to arid conditions in addition to introduction history and germplasm exchange.

Finally, the reduced 150-SNP fingerprint (**Figures 8**, **9**) provides a minimalist, operational tool for routine identity, purity testing, and curation in genebanks and nurseries. Genome-wide spacing and high minor-allele informativeness are the key attributes of robust fingerprint sets in clonally propagated crops. This SNP-based genomic fingerprinting complements plastid DNA barcodes used for rapid botanical identification (e.g., rbcL and matK), adding cultivar-scale resolution that chloroplast markers typically lack within-species contexts (Group1 et al., 2009). Such targeted, high-throughput SNP toolkits are increasingly recommended in perennial fruit programs as they integrate with genomic resources and domestication insights from tree crops, enabling varietal authentication, pedigree resolution, and cluster-aware sampling strategies for association studies and pre-breeding (Wang et al., 2023). An important limitation of the present fingerprinting panel is that it was selected and validated within the same study panel. Although the 150 SNPs were chosen using objective criteria, including high informativeness and broad chromosomal distribution, within-dataset validation does not fully exclude the possibility of overfitting. External validation was not feasible in the present study because no publicly available mango whole-genome resequencing or high-density genotyping dataset with overlapping markers and comparable accession representation was available for direct testing. Accordingly, the current results should be interpreted as strong internal validation of the panel’s discriminatory utility, while independent cross-dataset validation remains an important priority for future work as larger mango genomic reference panels become available.

More broadly, our results fit a general picture for long-lived, vegetatively propagated fruit trees: shallow domestication bottlenecks relative to annuals, geographically mediated structure with admixture, and chromosome-scale variation shaped by historic genome evolution. Mango provides a clear example, but convergent evidence spans citrus, apple, grapevine, peach, pear, and banana, as reference genomes and resequencing maps have clarified the genomic footprints of domestication and diversification in tree crops (Wu et al., 2018). Methodologically, pairing ADMIXTURE with PCA on LD-pruned SNPs (**Figure 5**–**7**), together with rigorous QC (**Figure 1**), reflects current best practice for population inference in crops. These choices reduce artefacts, stabilize K selection, and improve the interpretability of structure and phylogeny when viewed in a genome-scale context (**Figures 4**, **6**–**8**) (Alexander et al., 2009).

## 5 Conclusions

Whole-genome resequencing of 60 mango accessions indicated substantial genome-wide diversity, supported by 699,430 LD-pruned SNPs with a mean MAF = 0.2151, a mean PIC = 0.2463, and a mean expected heterozygosity (He) of 0.3003. Because all analyzed accessions originated from the Jazan region of Saudi Arabia, these findings should be interpreted as a regional genomic reference for Jazan mango germplasm rather than as a complete representation of mango diversity across Saudi Arabia. The population structure was resolved into three genetically differentiated ancestry groups under the K = 3 ADMIXTURE model. A comprehensive analysis of phylogeny, PCA, and admixture was conducted using 699,430 high-quality LD-pruned SNPs, followed by the development of a compact 150-SNP panel for precise cultivar identification and germplasm management. These results provide a valuable genomic reference for mango accessions of diverse origin and deliver robust resources for high-resolution fingerprinting, population structure characterization, and the preservation of genetic diversity. In addition, the regional structure observed in the Jazan panel provides an initial framework for future studies investigating the genomic basis of mango adaptation to hot, arid production systems in Saudi Arabia. For breeding, the three well-differentiated genetic groups and the genome-wide SNP dataset can guide the strategic selection of genetically distant parents, the design of mapping populations, and the implementation of marker-assisted and genomic selection for key traits adapted to arid environments. For conservation, the clear resolution of unique and admixed lineages, together with the SNP-based barcode, enables the detection of redundancy, the protection of rare or underrepresented gene pools, and the rational prioritization of accessions for *ex situ* and *in situ* conservation programs. The dataset and analytical workflow outlined here will, therefore, facilitate future genome-wide association studies, trait mapping, and the development of improved mango cultivars, as well as informed germplasm management in tropical and subtropical production areas.

## Data Availability

The datasets generated and analyzed during this study are available in Figshare at https://doi.org/10.6084/m9.figshare.31925922. Due to institutional data-sharing policies of the Ministry of Environment, Water and Agriculture (MEWA), the data are not publicly accessible. Access to the data can be provided by the corresponding author upon reasonable request and with permission from the relevant authority.

## Supplementary material

Supplementary Figure 1 A map showing the sampling location of all analyzed accessions in the Jazan region of southwestern Saudi Arabia.

Supplementary Figure 2 ADMIXTURE bar plots showing the inferred genetic structure of 64 mango accessions at K = 2–10.

Supplementary Figure 3 Sample-level heterozygosity was assessed using the inbreeding coefficient (F) across the mango accessions retained after missingness filtering. The two excluded outliers, M30 and M51, are highlighted in red. Most accessions cluster near zero, whereas these two samples show extreme deviations and were removed from downstream analyses.

Supplementary Figure 4 Genome-wide linkage disequilibrium (LD) decay based on the full, non-LD-pruned SNP dataset.

Supplementary Figure 5 Cross-validation errors from ADMIXTURE for K = 2–5 based on the LD-pruned SNP dataset. The minimum CV error was observed at K = 3, supporting this value as the best-fitting model, although K = 2 and K = 4 showed only slightly higher errors.

Supplementary Figure 6 Per-chromosome nucleotide diversity (π) across the 20 mango chromosomes.

Supplementary Figure 7 Mango accessions 2D barcode.

Supplementary Table 1 Passport data (local name, origin, and collection site) for the 64 mango accessions from the Jazan region.

Supplementary Table 2 Sequencing yield, mapping statistics, and SNP calling summary for each accession.

Supplementary Table 3 List of 699,430 high-quality SNPs used in population genetic analyses.

Supplementary Table 4 List of the 150 SNPs included in the fingerprinting panel, with chromosome position and PIC values.

Supplementary Table 5 Cluster-specific observed heterozygosity (Ho), expected heterozygosity (He), pairwise weighted FST, and nucleotide diversity (π) for the 60 curated accessions.

Supplementary Table 6 Pairwise genetic distances among mango accessions based on LD-pruned SNPs.

File S1 Genotype matrix (VCF format) for the filtered SNP dataset used in this study.
